# Long-term predictivity of early neurological assessment and developmental trajectories in low-risk preterm infants

**DOI:** 10.3389/fneur.2022.958682

**Published:** 2022-09-27

**Authors:** Daniela Dicanio, Giulia Spoto, Angela Alibrandi, Roberta Minutoli, Antonio Gennaro Nicotera, Gabriella Di Rosa

**Affiliations:** ^1^Unit of Child Neurology and Psychiatry, Department of Human Pathology of the Adult and Developmental Age, “Gaetano Barresi” University of Messina, Messina, Italy; ^2^Department of Economics, University of Messina, Messina, Italy

**Keywords:** general movements assessment, hammersmith infant neurological examination, griffiths mental development scales, low-risk preterm, prematurity, preterm

## Abstract

Prematurity represents 10.6% of all births, and although preterm infants usually show adequate neurodevelopmental outcomes, some may develop significant and long-lasting neurological sequelae. Many studies have analyzed predictive factors for developing severe neurodevelopmental impairments (cerebral palsy, other motor and socio-relational disorders such as autism). In this study, 148 preterm infants were enrolled to investigate the neurodevelopmental trajectories in a population of low-risk premature infants using standardized assessment methods. Significant correlations were found between the general movements, the Hammersmith Infant Neurological Examination, and the Griffiths Mental and Development Scales. Moreover, this study showed their validity and predictivity for adverse neurodevelopmental outcomes even in low-risk infants.

## Introduction

Preterm birth is defined by the WHO as a live birth before 37 completed weeks of gestation and, recently, it has been considered a matter of utmost importance, representing the leading cause of death in children aged under 5 years (1). The exact prevalence of prematurity is difficult to estimate due to the lack of systematic recording of these data. However, an increase in preterm births has been recently recorded in most countries, and preterm birth rates in the last decade amount to 10.6% of all births (1, 2). Multiple classification systems on preterm birth have been developed to guide research on determinant causes, identify risk populations, implement prevention strategies, increase preterm birth surveillance, and standardize local and international data measurements. The most common preterm classification systems are based on gestational age (GA; including extremely preterm, very preterm, moderate preterm, and late-preterm infants), mode of delivery (spontaneous vs. provider-initiated), etiology, or pathophysiological pathways (3). Many risk factors have been related to preterm birth, including maternal causes (alcohol, drugs, diabetes, and nephropathy), pregnancy complications, multiple births, and intrauterine growth retardation (4). However, even though many socio-demographic, nutritional, medical, obstetrical, and environmental factors have been associated with an increased risk of spontaneous preterm birth, its precise etiology remains unclear (3).

Prematurity is considered a risk factor both for short- and long-term complications. Short-term complications usually occur in the first days of life. They include neonatal respiratory conditions (such as respiratory distress syndrome and bronchopulmonary dysplasia), necrotizing enterocolitis, sepsis, neurological conditions (including periventricular leukomalacia, intraventricular hemorrhage, hypoxic-ischemic encephalopathy, cerebral palsy, and seizures), feeding difficulties, and visual and hearing problems (2, 3, 5, 6). Conversely, preterm birth is also related to altered neurodevelopmental outcomes. Although preterm infants usually show good neuropsychological development, some of them, especially very preterm ones, may develop significant and long-lasting neurological sequelae (7, 8). Healthcare advances have increased survival rates for extremely preterm children and reduced the risk of significant motor impairment (i.e., cerebral palsy). However, the neonatal intensive care unit (NICU) environment, which is very different from the intrauterine one, exposes them to risks of long-term effects (9, 10). As a result, the number of preterm infants with poor neurodevelopmental outcomes did not drop as intended. Preterm infants are more frequently admitted to hospitals and present a higher risk of behavioral, social–emotional, and learning difficulties in childhood compared to term newborns (3, 7, 11, 12).

Recently, research focused on finding the best-validated diagnostic tools to allow an early diagnosis and provide timely intervention in high-risk infants to minimize significant impairments, such as cerebral palsy (13). However, nearly 85% of preterm births occur in the late-preterm period (34^+0^-36^+6^ weeks). These children are considered a low-risk population because they appear less likely to develop pathological developmental trajectories compared to extremely and very preterm infants (2, 4, 11, 14). Nevertheless, late-preterm babies have significantly higher risks of adverse outcomes compared to term newborns and may show various neurodevelopmental problems, such as neurocognitive and social interaction problems (3, 15). Late-preterm children are often cared for like their term counterparts. However, this population has a higher rate of speech disorders, attention deficit hyperactivity disorder, autism spectrum disorder, behavioral problems, specific learning disorder, developmental delay or intellectual disability, and psychiatric disorders (15–19).

Therefore, prematurity should be considered a risk factor for poor neurodevelopmental outcomes even in the absence of significant impairments (i.e., brain injury) and late-preterm babies should not be excluded from the usual follow-up programs (20, 21). Moreover, standardized and predictive evaluation systems are urgently required to diagnose pathological development trajectories early. The aims of our study are (1) to investigate the neurodevelopmental trajectories in a population of low-risk premature infants using standardized assessment methods (General Movements Assessment, Hammersmith Infant Neurological Examination, and Griffiths Mental Developmental Scale) and (2) to verify their validity and predictivity for poor neurodevelopmental outcomes even in low-risk infants.

## Methods

Caucasian preterm infants (born before 37 weeks of pregnancy) were recruited at the Unit of Child Neurology and Psychiatry at the University Hospital “G. Martino” of Messina, Italy, between September 2018 and October 2020, and were enrolled in a follow-up prospective research program.

The population has been selected according to the following inclusion criteria: (1) GA comprised between 28^+0^-36^+6^ weeks; (2) weight appropriate for GA, considering appropriate a weight between 10th and 90th percentile; (3) normal cranial ultrasound (cUS) or mild abnormal findings such as transient flares (lasting < 2 weeks) or germinal layer hemorrhages grade 1 (IVH I) according to Volpe (22); (4) absence of congenital malformations; (5) absence of neurosensory deficits.

According to GA, the preterm cohort was divided into two subgroups: early preterm (GA between 28^+0^ and 33^+6^ weeks) and late preterm (GA between 34^+0^ and 36^+6^ weeks).

Medical history was collected considering pre-, peri-, and postnatal variables for all patients. In particular, the following variables have been considered: placental abruption, premature rupture of membranes, emergency cesarean section, APGAR score at the first and fifth minute of life, birthweight, neonatal intensive care unit recovery, hypoglycemia, neonatal sepsis, neonatal seizures, acute respiratory distress syndrome, bronchopulmonary dysplasia, pneumothorax, invasive and non-invasive ventilation, steroid treatment, caffeine treatment, retinopathy of prematurity, and blood transfusion. Altered sleep–wake cycle, gastrointestinal disorders (gastroesophageal reflux disease and difficulty in sucking), and pathological otoacoustic emissions were also considered.

To identify the presence of cerebral lesions, cUS evaluations were performed on preterm infants within the first week of life and always around term-equivalent age. If an abnormal finding was recorded, weekly monitoring was performed. According to Volpe (22), transient flares (lasting <2 weeks) were considered normal findings, while persistent flares and IVH1 were considered pathological.

As part of the follow-up program, a video recording of General Movements (GMs) was performed during the visit. The subjects were recorded during the inter-feeding time, in the supine position, in a nappy. The videos were analyzed by a certified evaluator (GD) according to the standard methodological principles of Prechtl's method (23).

General Movements are part of a complex spontaneous movement repertoire that already appears in early fetal life. They are fluent movements that occur frequently, have a gradual beginning and end, and involve the whole body in variable sequences (24). Two distinct GM patterns can be observed from term age onward: Writhing movements and Fidgety movements (FMs). Writhing movements are present from term age up to about 9 weeks. FMs appear at ~6 weeks and can be present up to about 20 weeks, when goal-directed movements start to prevail (25, 26). During the Writhing period, movement repertoire is classified and ranked for statistical analysis as (1) normal (N), (2) sub-optimal (poor repertoire), and (3) abnormal (chaotic or cramped synchronized). Similarly, during the Fidgety period, movements are classified and ranked as (1) normal FMs, (2) abnormal FMs, or (3) absent FMs. In the present study, GM Assessment (GMA) was performed for each infant during the Fidgety period, and the results were classified and ranked for statistical analysis either as (1) normal FMs, (2) abnormal FMs, or (3) absent FMs.

The Hammersmith Infant Neurological Examination (HINE) is a simple and scorable neurological assessment for infants between 2 and 24 months of age. It comprises 26 items that evaluate cranial nerves, posture, movements, tone, and reflex reactions. Each item is given a score from zero to three, and the sum of the individual scores determines a global score that can range from a minimum of 0 to a maximum score of 78. Based on the frequency distribution of the scores in the normal population, an optimality score has been developed for research purposes. Global scores are reported as optimal if they are ≥67 at 3 months, ≥70 at 6 months, and ≥73 at 9 to 12 months (4, 24, 27). HINE was performed at 3-, 6-, and 9-month corrected age (CA) by the same physician (GD); the items were scored separately, and the global score was calculated.

The neurodevelopmental evaluation was performed between 12 and 36 months of life with the validated Italian translations of the Griffiths Mental Development Scales Revised (GMDS-R) for children aged 0–24 months and the Griffiths Mental Development Scales Extended Revised (GMDS-ER) for children 2–8 years of age (28). The assessment consists of five scales that investigate locomotor, personal–social, hearing and language, eye–hand coordination, and performance domains. The GMDS-ER also comprises a Practical Reasoning scale that has not been administered in our study since it can be applied from 3 years of age. Standardized general development quotient (mean 100, SD 12) and sub-quotients (mean 100, SD 16) were scored. According to Battaglia and Savoini (28), general development quotient ≥88 and sub-quotients ≥84 characterize typical development, whereas general development quotient <88 and sub-quotients < 84 identify a developmental delay. Normative data for the Italian version of the GMDS pertain to the 1996 UK version revised by Huntley (28).

The general development quotient and the sub-quotients were calculated for CA and chronological age. The scales were always performed by the same examiner (RM).

Data were securely stored and managed using an electronic data capture tool hosted at Unit of Child Neurology and Psychiatry at the University Hospital “G. Martino” of Messina. Missing demographic data and other clinical characteristics of the enrolled subjects were also collected using telephonic interviews. Data on family and personal medical history, presence of comorbidities, and drug use were also collected.

The Institutional Review Board of the University of Messina approved the study. A written informed consent was obtained from the parents of the infants.

### Statistical analysis

Categorical variables were expressed as absolute and percentage frequencies and numerical variables as mean and standard deviation.

The Kolmogorov–Smirnov test was applied to verify the normality of the distribution of the variables examined. The application of the test made it possible to ascertain the condition of normality for most of the variables considered; consequently, it was decided to use the parametric approach for the statistical analysis of the data.

Student's *t*-test was applied to identify the existence of statistically significant differences between two groups of patients (early preterm vs. late preterm) regarding the numerical variables, with reference to the categorical variables; on the contrary, the comparison between the two groups was carried out using the chi-square test.

The possible correlations between all the variables were also investigated in the whole group by stratifying by group (early preterm and late preterm) using the Spearman test.

The predetermined significance level is α = 0.050; therefore, *p*-value < 0.050 for two-tailed tests was considered statistically significant.

Multiple linear regression models were estimated to identify significant predictors of the interest outcomes, such as general development quotient, locomotor scale, personal–social scale, hearing and language scale, eye–hand coordination scale, and performance scale, all considered for chronological age. Tested covariates were as follows: GMs, posture subsection (in the three timepoints), movement subsection (in the three timepoints), tone subsection (in the three timepoints), reflex reaction subsection (in the three timepoints), cranial nerve subsection (in the three timepoints), developmental milestones, and birthweight.

The statistical analysis was performed using SPSS software version 22.0 (IBM SPSS Statistics, Chicago, IL, USA).

## Results

A total of 148 infants were enrolled in the study, 85 males (57.4%) and 63 females (42.6%). The mean GA of the entire group was 33.2 ± 2.1 weeks, and the average birth weight was 1,944 ± 565 g. Pre-, peri-, and postnatal risk factors and cUS findings were collected for all the enrolled patients. About 10.1% of the total group have a history of placental abruption during the pregnancy, with a significantly higher percentage in the early preterm group than the late-preterm group (15.1% vs. 3.2%; *p*-value = 0.018). The early preterm group had a mean birthweight of 1670.4 ± 439.1 g, while the late-preterm babies had a mean birthweight of 2319.1 ± 502.9 g. The mean 1-min and 5-min Apgar scores resulted significantly lower in the early preterm group than in the late-preterm group, being 7.0 ± 1.6 vs. 8.1 ± 1.4 (*p*-value < 0.001) and 8.4 ± 0.9 vs. 9.1 ± 0.9 (*p*-value < 0.001), respectively. In 94.6% of the cases, a neonatal intensive care unit recovery was required, particularly in 97.7% of the early preterm babies and 90.3% of the late-preterm group. Eighty-eight patients (59.5% of the total group) had acute respiratory distress syndrome (ARDS). It occurred more frequently in the early preterm group when compared to late-preterm group (71 vs. 43.5%; *p*-value < 0.001), requiring in 65.5% of cases a non-invasive ventilation (74.4% of the early preterm babies vs. 53.2% of the late-preterm ones; *p*-value = 0.008) and in 19.6% of cases an invasive ventilation (25.6% of the early preterm group vs. 11.3% of the late-preterm group; *p*-value = 0.031).

Moreover, the early preterm group more frequently received blood transfusions (32.5 vs. 13.0%; *p*-value = 0.007) compared to the late-preterm group.

CUS showed normal findings in 62.8% of the total group of preterm infants and, in particular, in 66.3% of the early preterm babies and 58% of late-preterm ones. Transient flares were observed in 24.3% of the total group, 22.1% of the early preterm group, and 17.7% of the late-preterm group. In comparison, IVH1 was observed in 6.1% of the total group, 8.1% of the early preterm group, and 3.2% of the late-preterm group. No statistically significant differences regarding the cUS findings were observed between the two groups.

The features, pre-, peri-, and postnatal risk factors, and cUS findings for all enrolled patients and the two subgroups (early preterm and late preterm) are summarized in Table 1.

**Table 1:** Clinical characteristics of the preterm population.

	**Total population** **(28^+0^-36^+6^ weeks)** ***n*** **= 148**	**Early preterm** **(28^+0^-33^+6^ weeks)** ***n*** **= 86**	**Late preterm** **(34^+0^-36^+6^ weeks)** ***n*** **= 62**	**Difference between early and late preterm** **(*p-value*)**
Sex	**M** 85 (57.4%) **F** 63 (43.6%)	**M** 46 (53.5%) **F** 40 (46.5%)	**M** 39 (62.9%) **F** 23 (42.6%)	M 0.255 F 0.639
Birthweight	1,944.5 ± 565.7 g	1,670.4 ± 439 g	2,319.1 ± 502.9 g	**< 0.001**
1-min Apgar score	7.5 ± 1.6	7.0 ± 1.7	8.1 ± 1.4	**< 0.001**
5-min Apgar score	8.7 ± 1.0	8.4 ± 1.0	9.1 ± 1.0	**< 0.001**
Placental abruption	15 (10.1%)	13 (15.1%)	2 (3.2%)	**0.018**
Premature rupture of membranes	8 (5.4%)	6 (7.0%)	2 (3.2%)	0.315
Emergency cesarean section	105 (70.9%)	65 (75.6%)	40 (65.5%)	0.181
NICU recovery	140 (94.6%)	84 (97.7%)	56 (90.3%)	0.050
Hypoglycemia	10 (6.7%)	7 (8.1%)	3 (4.8%)	0.430
Neonatal sepsis	13 (8.8%)	9 (10.5%)	4 (6.4%)	0.386
Neonatal seizures	5 (3.4%)	3 (3.5%)	2 (3.2%)	0.921
Acute respiratory distress syndrome	88 (59.5%)	61 (71.0%)	27 (43.5%)	**< 0.001**
Bronchopulmonary dysplasia	1 (0.7%)	1 (0.7%)	0 (0%)	0.511
Pneumothorax	19 (12.8%)	10 (11.6%)	9 (14.5%)	0.604
Non-invasive ventilation	97 (65.5%)	64 (74.4%)	33 (53.2%)	**0.008**
Invasive ventilation	29 (19.6%)	22 (25.6%)	7 (11.3%)	**0.031**
Steroid treatment	12 (8.1%)	13 (15.1%)	10 (16.1%)	0.869
Caffeine treatment	8 (5.4%)	6 (7.0%)	2 (3.2%)	0.315
Blood transfusion	36 (24.3%)	28 (32.5%)	8 (13.0%)	**0.007**
Retinopathy of prematurity	2 (1.3%)	1 (1.2%)	1 (1.6%)	0.837
Altered sleep-wake cycle	7 (4.7%)	6 (7.0%)	1 (1.6%)	0.128
Gastrointestinal disorders	20 (13.5%)	9 (10.5%)	11 (17.7%)	0.208
Pathological otoacoustic emissions	3 (2.0%)	3 (3.5%)	0 (0%)	0.138
Normal cranial ultrasound	93 (62.8%)	57 (66.3%)	36 (58.0%)	0.304
Transient flares	36 (24.3%)	19 (22.1%)	11 (17.7%)	0.512
IVH 1	9 (6.1%)	7 (8.1%)	2 (3.2%)	0.219

Data in bold are statistically significant.

### Neurodevelopmental assessments

General Movements assessment was performed for all groups of infants during the Fidgety period. HINE was performed at 3-, 6-, and 9-month CA on 67.6, 52, and 41% of the patients, respectively. Sixty-three infants (42.6% of the total group) were subsequently evaluated with GMDS-R (82.5%) and GMDS-ER (17.5%). At the evaluation, the early preterm group had a mean of the chronological age of 18.4 ± 5.8 months (mean of corrected age 16.3 ± 5.8 months), while the late-preterm group had a mean chronological age of 18.7 ± 6.6 months (mean of corrected age 17.3 ± 6.6 months). During the follow-up, seven of the infants (4.7%), specifically five (5.8%) from the early preterm group and two (3.2%) from the late-preterm group, developed motor impairments, and they were excluded from further statistical analyses.

General Movements were assessed in each infant during the Fidgety period: 68.1% of the population showed normal FMs (64.2% of the early preterm group vs. 73.3% of the late-preterm group), 27.7% presented abnormal FMs (29.6 vs. 25%), while the FMs were absent in 4.2% of the total group (6.2 vs. 1.7%). The differences between the two groups were not statistically significant. Data are summarized in Table 2.

**Table 2:** General movement scores (Fidgety movement scores).

	**Total population** ***N =*** **141 (% intra-group)**	**Early preterm** ***N*** **= 81** **(% intra-group)**	**Late preterm** ***N*** **= 60** **(% intra-group)**	**Difference between early and late preterm** **(*p*-value)**
Normal FMs	96 (68.1%)	52 (64.2%)	44 (73.3%)	0.253
Abnormal FMs	39 (27.7%)	24 (29.6%)	15 (25%)	0.547
Absent FMs	6 (4.2%)	5 (6.2%)	1 (1.7%)	0.194

Hammersmith Infant Neurological Examination was administered to 94 children (66.7% of the total group) at 3 months CA, to 73 infants (51.8%) at 6 months CA, and 63 patients (44.7%) at 9-month CA. HINE scores are summarized in Table 3. No significant differences emerged between the two groups, considering both the global and subsections scores, though the early preterm group obtained slightly higher scores than the late-preterm group in all the evaluations.

**Table 3:** Hammersmith infant neurological examination (HINE) scores.

	**Cranial nerves** **Mean - SD**	**Posture** **Mean - SD**	**Movements** **Mean - SD**	**Tone** **Mean - SD**	**Reflex reactions** **Mean - SD**	**Global score** **Mean - SD**
**3 months**						
Total group	14.3 ± 1.7	9.8 ± 3.8	5.2 ± 1.3	20.0 ± 3.1	6.2 ± 2.2	55.6 ± 7.6
Early preterm	14.4 ± 1.5	9.8 ± 3.8	5.2 ± 1.3	20.3 ± 2.8	6.3 ± 2.0	56.1 ± 6.8
Late preterm	14.1 ± 1.8	9.8 ± 3.9	5.2 ± 1.3	19.7 ± 3.5	6.1 ± 2.4	54.9 ± 8.5
Term infants^*^	13^*^	14^*^	5^*^	18^*^	8^*^	64 (63–66)^*^
**6 months**						
Total group	14.8 ± 0.6	12.3 ± 3.7	5.6 ± 1.1	21.5 ± 2.5	10.3 ± 3.1	64.4 ± 6.6
Early preterm	14.8 ± 0.5	12.4 ± 3.6	5.5 ± 1.1	21.8 ± 1.6	10.3 ± 3.2	64.9 ± 6.3
Late preterm	14.8 ± 0.6	12.0 ± 4.0	5.6 ± 0.9	21.1 ± 3.5	10.2 ± 3.0	63.8 ± 7.0
Term infants^*^	14^*^	14^*^	5^*^	20^*^	10^*^	68 (67–70)^*^
**9 months**						
Total group	14.9 ± 0.5	15.9 ± 2.6	5.7 ± 0.8	21.3 ± 2.2	11.9 ± 3.1	69.7 ± 6.0
Early preterm	14.9 ± 0.5	16.0 ± 2.2	5.7 ± 0.8	21.3 ± 1.7	12.4 ± 2.8	70.5 ± 4.9
Late preterm	14.9 ± 0.4	15.5 ± 3.0	5.7 ± 0.8	21.2 ± 2.9	11.1 ± 3.4	68.4 ± 6.4
Term infants^*^	14^*^	15^*^	5^*^	20^*^	11^*^	72 (70–73)^*^

Median and range of global and subsection scores in total group, early preterm, late-preterm, and term* born infants.

* Data from the literature (29).

A significant correlation between GMs scores and HINE global scores at 3-, 6-, and 9-month CA evaluations (*p*-value = 0.004, <0.001, and <0.001, respectively) was evidenced. Better GMA significantly correlated with higher scores in the subsection movements (*p*-value < 0.001) at 3-, 6-, and 9-month CA evaluations, with the subsection posture at 6- and 9-month CA evaluations (*p*-value = 0.011 and <0.001, respectively), with the subsection tone at 3- and 6-month CA evaluations (*p*-value = 0.013 and 0.032, respectively), and with the subsection reflex reactions at 3- and 9-month evaluations (*p*-value = 0.009 and <0.001, respectively).

GMDS was performed in 63 infants (44.7% of the total group) between 12 and 36 months (mean of chronological age 18.5 ± 6.1 months/mean of corrected age 16.7 ± 6.2 months). Considering the chronological age, no significant differences were detected between the scores of the two groups: The mean general development quotient appeared to be slightly below the standard both in the early (86.5 ± 14.4) and the late-preterm babies (87.7 ± 17.5), whereas the subscales quotients resulted within the normal range in both groups. The only exception is represented by the hearing and language scale, in which the mean score resulted within the standards in the late-preterm babies (89.7 ± 24.2) and below the normal range in the early preterm infants (78.4 ± 20.4) and the total group (82.3 ± 22.6). All the quotients were assessed within the normal range of scores when assessed by CA, and data are summarized in Table 4.

**Table 4:** Griffiths Mental Development Scale (GMDS) scores.

	**Chronological age** **Mean ±SD**	**Corrected age** **Mean ± SD**	**EP** **chronological age** **Mean ±SD**	**LP chronological age** **Mean ±SD**	***p*** **-Value**	**EP** **correct age** **Mean ±SD**	**LP** **correct age** **Mean ±SD**	***p*** **-Value**
General development (Q)	**87.0 ±15.7**	98.4 ± 19.2	**86.5 ±14.4**	**87.7 ±17.6**	0.766	99.3 ± 17.7	97.2 ± 21.4	0.687
Locomotor (Q)	91.3 ±18.4	116.8 ± 106.2	91.0 ± 16.2	91.6 ± 21.2	0.909	104.0 ± 18.5	134.5 ± 162.7	0.350
Personal-social (Q)	88.3 ± 17.7	99.4 ± 21.3	88.0 ± 16.6	88.7 ± 19.3	0.890	100.7 ± 20.4	97.6 ± 22.9	0.586
Hearing and language (Q)	**82.3 ±22.6**	93.3 ± 25.3	**78.4 ±20.4**	89.7 ± 24.2	0.055	90.0 ± 24.3	97.8 ± 26.4	0.239
Eye-hand coordination (Q)	85.8 ± 17.2	96.8 ± 21.2	86.8 ± 15.0	84.6 ± 20.1	0.633	99.2 ± 18.3	93.5 ± 24.7	0.323
Performance (Q)	87.6 ± 19.0	98.9 ± 23.2	90.0 ± 19.2	84.5 ± 18.8	0.262	103.1 ± 23.2	93.0 ± 22.3	0.087

Data in bold resulted below the normal range.

Q, quotient; EP, early preterm group; LP, late-preterm group.

A significant correlation (*p*-value = 0.029) between GMA and the eye–hand coordination scale of the GMDS was detected.

**Figure 1:**
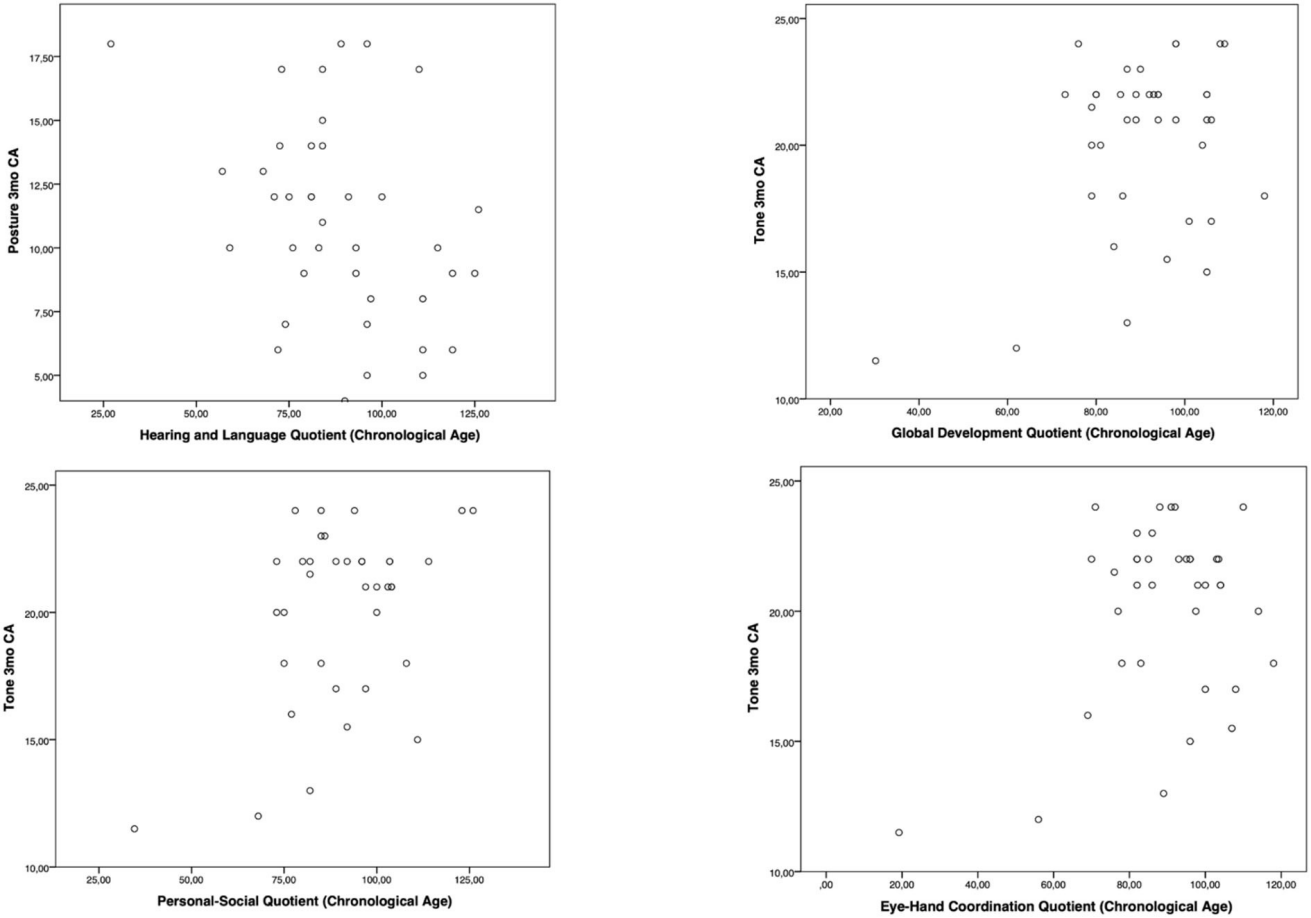
Significant correlations between GMDS and HINE at 3-month CA evaluation.

**Figure 2:**
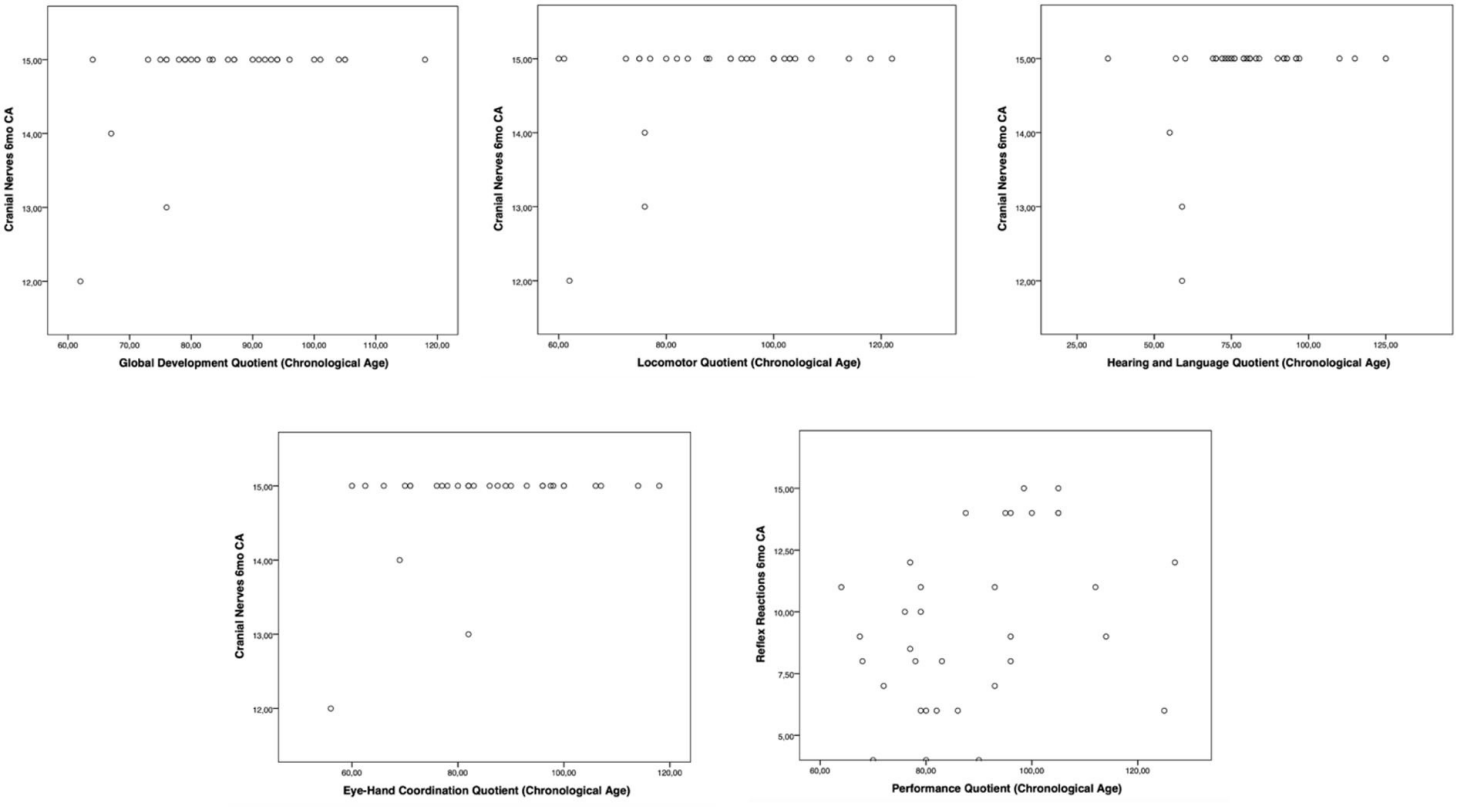
Significant correlations between GMDS and HINE at 6-month CA evaluation.

**Figure 3:**
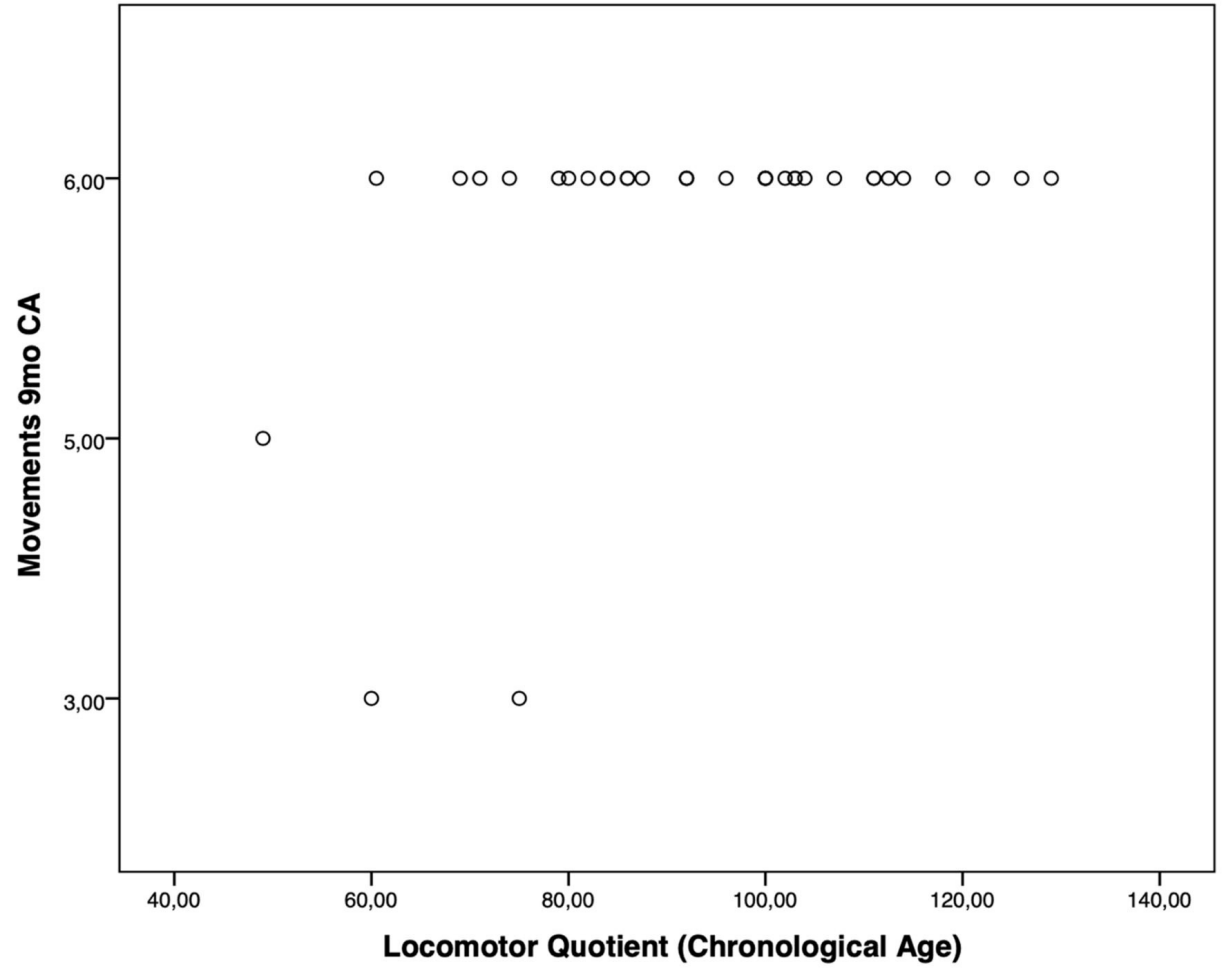
Significant correlations between GMDS and HINE at 9-month CA evaluation.

Moreover, the HINE subsections revealed several significant correlations with the GMDS general development score and the different scales, considering the chronological age. In particular, at the 3-month CA evaluation, higher scores of the subsection posture presented a significant correlation with the hearing and language scale (*p*-value = 0.019); the subsection tone had a significant correlation with higher scores of general development score (*p*-value = 0.019) and with personal–social (*p*-value = 0.004) and eye–hand coordination (*p*-value = 0.040) scales (Figure 1). Considering the 6-month CA evaluation scores, a significant correlation emerged between the subsection cranial nerves and the general development score (*p*-value = 0.011), the locomotor scale (*p*-value = 0.016), the hearing and language scale (*p*-value = 0.041), and the eye–hand coordination scale (*p*-value = 0.039); the subsection reflex reactions had a significant correlation with the performance scale (*p*-value = 0.039) (Figure 2). The subsection movements at 9-month CA evaluation had a significant correlation with the locomotor scale (*p*-value = 0.010; Figure 3).

Multiple linear regression showed significant correlations between the locomotor scale, the posture at 6 months (CA), and the movement subsection at 3- and 6-month (CA) evaluations. Moreover, the subsection posture at the 6-month CA evaluation significantly correlated with the personal–social scale and the general development quotient. Multiple linear regression data are shown in Table 5.

**Table 5:** Multiple linear regression analysis.

**GD (Q)**	***B***	***SE***	**Beta**	***p*** **-Value**	**95% CI of** ***B***		
Constant	61.438	11.346		< 0.001	35.771	87.104	*R* = 0.686 *R*^2^ = 0.471 Adj. *R* = 0.412
Posture at 6 mo CA	2.501	0.884	0.868	**0.020**	0.503	4.500	

Excluded variables (stepwise): GMs; birthweight; posture at 3- and 6-mo CA; movements at 3-, 6-, and 9-mo CA; tone at 3-, 6-, and 9-mo CA. Data in bold are statistically significant.

**Table:** 

***L*** **(Q)**	***B***	**SE**	**Beta**	***p*** **-Value**	**95% CI of** ***B***		
Constant	113.673	2.811		0.001	101.578	125.768	*R* = 0.999 *R*^2^ = 0.998 Adj. *R* = 0.994
Movements at 6 mo CA	−5.788	0.302	−0.702	**0.003**	−7.086	−4.489	
Movements at 3 mo CA	2.497	0.307	0.364	**0.015**	1.178	3.816	
Posture at 6 mo CA	0.669	0.155	0.198	**0.050**	0.003	1.335	

Excluded variables (stepwise): GMs; posture at 3- and 9-mo CA; tone at 3-, 6-, and 9-mo CA; reflex reactions at 3- and 9-mo CA; sitting position; standing position; deambulation.

**Table:** 

**PS (Q)**	***B***	**SE**	**Beta**	***p*** **-Value**	**95% CI of B**		
Constant	54.492	10.699		0.001	30.289	78.696	*R* = 0.755 *R*^2^ = 0.570 Adj. *R* = 0.523
Posture at 6 mo CA	2.879	0.833	0.755	**0.007**	0.995	4.764	

Excluded variables (stepwise): GMs; posture at 3- and 9-mo CA.

GD, general development; Q, quotient; Std, standard; CI, confidence interval; mo, months old; CA, corrected age; L, locomotor scale; PS, personal–social scale.

## Discussion

Nowadays, prematurity is considered a risk factor for both major and minor neuropsychological sequelae, driving the need for effective assessments that may be highly predictive of developmental outcomes in early life. GMA and HINE have been proven to be highly accurate tools, having together with neuroimaging, a sensitivity of >97% and a specificity of >97% in detecting significant neurological impairments (i.e., cerebral palsy). Therefore, they are considered the gold-standard tools in early diagnosis of children at high risk for neurological sequelae (13, 24).

However, the interest in the early prediction of minor outcomes has increased, posing a new challenge to the scientific community (10, 29–31).

This study aimed to analyze the predictivity of standardized early neurological assessments in a population of low-risk babies and to identify precocious neurological predictors of adverse neurodevelopmental outcomes.

In a study on the ontogeny of FMs, Ferrari et al. (32) reported jerky and larger FMs in children assessed before 12 weeks of age, while they presented smoother and smaller movements in the following weeks, establishing that the best period to assess the FMs is within 12–16^+6^ weeks. However, Kwong et al. (33) observed that FMs are still maturing within the 12–16^+6^ weeks window in preterm children, presenting a higher percentage (23%) of absent/abnormal FMs than the term-born low-risk population. In this cohort of patients, 15% of patients presenting abnormal/absent FMs at 12 weeks showed normal FMs at the 14-week observation, indicating a favorable GMs trajectory (33).

In our clinical follow-up, a single GMA is performed at 3-month CA, detecting normal FMs in 68.1%, abnormal FMs in 27.7%, and absent FMs in 4.2% of the patients. These data represent an unexpected result since in the low-risk term-born population, the presence of absent/abnormal FMs stands at 2–3% (33). The high percentage of abnormal/absent FMs reported in our population could be related to the single observation at 3-month CA in those children in which FMs are emerging or have not emerged yet. A limitation of our study, which would have provided a better estimate of the percentage of absent/abnormal FMs, was that we could not evaluate FMs in a second evaluation and did not perform a systematic evaluation of the GMs' trajectory.

Nevertheless, we found a significant correlation between the GMs scores and HINE global scores at 3-, 6-, and 9-month corrected age, confirming the efficacy of the integrated use of these two methods instead of the single assessment. Indeed, they evaluate different constructs and do not provide similar prognostic information (34, 35).

To the best of our knowledge, no studies compared the GMA to the subsections of the HINE. Nevertheless, the significant correlations between GMs scores and the subsections movements at 3-, 6-, and 9-month CA evaluations, posture at 6- and 9-month CA evaluations, tone at 3- and 6-month CA evaluations, and reflex reactions at 3- and 9-month CA evaluations represent an interesting data. It remarks the assumption that spontaneous movements such GMs are necessary for developing an appropriate quality of movements, posture, and tone during the first year of age. During infancy, the development of posture and motility is tightly entangled: from 1 month of age, the baby begins to make some direction-specific movements allowing it to develop the postural control needed to achieve the reaching skill in the subsequent months; in fact, these direction-specific postural adjustments have an innate origin (36). This correlation has an important significance, especially considering that GMA is the first evaluation that provides us with information on the motor development trajectory. Moreover, movements, posture, and tone are finely controlled by encephalic structures and alterations detected during these early assessments may act as red flags for minor lesions of the central nervous system that frequently occur in preterm babies, even the low-risk ones (37, 38).

Regarding the HINE assessment, when compared to full-term infants (27, 29), our sample cohort showed lower global scores at 3-, 6-, and 9-month CA evaluations (see Table 3). These data confirm the well-known notion that preterm infants differ greatly from their term counterparts during the first year of life and follow a specific developmental trajectory, probably due to brain immaturity (4, 21). On the contrary, the preterm infants in our study also showed slightly lower global scores when compared to low-risk preterm infants. In a recent study, Romeo et al. (31) established cutoff scores at 58, 64, and 69 for typical/mildly delayed performance at 3-, 6-, and 9-month CA, respectively. This difference may be ascribed to the reduced sample of our study (148 vs. 1229 preterm babies) or their younger mean age at birth (33.2 vs. 35.3 weeks PMA). However, even compared to a similar sample of very preterm infants (148 vs. 174), our study reports slightly lower global scores, especially at the 3-month CA evaluation (39). The posture subsection shows the greater difference, while the tone subsection displays a little discrepancy, and the cranial nerves, the movement, and the reflex reaction scores are consistent with the ones reported by Romeo et al. (39).

Data on GMDS correlations should be cautiously evaluated, since the assessment was performed only in 44.7% of the patients and during a wide age window assessment, representing a limitation of this study.

However, HINE subsections also showed several correlations with the GMDS. The subsection cranial nerves assessed at 6-month CA showed a significant correlation with the general development score, the locomotor scale, the eye–hand coordination, and the hearing and language scale. This confirms the data reported by Kyriakidou et al. (40), who conducted a study on 73 preterm babies, demonstrating that infants with lower scores in the cranial nerve subsection presented with lower motor scores. To date, the correlation between the cranial nerves and the hearing and language subsection has never been reported in the literature. Nevertheless, it represents an interesting data because precocious visual and auditory abilities underlie the development of communicative skills, and auditory function may influence early receptive language development in preterm babies (41).

At the 9-month CA evaluation, the subsection movements had a significant correlation with the locomotor scale, confirming that this is the most predictive subsection of the motor outcome (42). These data show that the early neurological evaluation, such as HINE, has a predictive value even compared with a development evaluation as the GMDS. Although a lower correlation of the HINE with 2-year outcome at 3 months has been reported, since HINE was originally validated from 12 months onward and some of the items are age-dependent, a significant correlation between the subsection tone at 3-month CA and the general development score of the GMDS is worth of mentioning: since preterm infants almost always show a reduction in tone when compared to full-term babies, this information proves that preterm babies with higher tone scores have a more similar outcome to full-term children (21, 29). The correlation found between the GMA and the eye–hand coordination scale of the GMDS indicates that GMs have a better predictive value of the motor outcome than the cognitive one.

Finally, important data are represented by the fact that the general development score assessed for CA is largely within the normal range, while it is slightly below the lower normal value if assessed for chronological age. These data indicate an important difference in the score calculation: In fact, it has been proven that preterm-born children often suffer from learning difficulties in school age and show impairments in all cognitive domains, leading to an intelligent quotient deficit (43). Therefore, assessing the quotients for chronological age would give the child a more realistic performance value than the score evaluated for the corrected age.

## Conclusion

Our study shows the neurodevelopmental trajectory in a population of preterm births using standardized assessments. Although significant risk factors related to severe prematurity certainly exist, the comparison between the two groups (early and late preterm) demonstrates a similar development pathway, not significantly influenced by their GA. This study underlines the importance of including even these low-risk populations in the follow-up programs to early identify infants at risk for adverse long-term impairments and introduce early intervention therapies for optimizing neurodevelopmental outcomes.

Therefore, it would be necessary to identify accurate tools to optimize the follow-up programs, allowing to continue monitoring low-risk premature infants up to 36 months of life. It would also be crucial to prove the predictivity of an early neurological assessment to identify abnormal development trajectories and realize an early intervention such as for high-risk babies.

## Data availability statement

The raw data supporting the conclusions of this article will be made available by the authors, without undue reservation.

## Ethics statement

The studies involving human participants were reviewed and approved by Institutional Review Board of University of Messina. Written informed consent to participate in this study was provided by the participants' legal guardian/next of kin.

## Author contributions

GR conceived, planned, and supervised the study and performed GMA and HINE. DD collected the data. DD and GS wrote the first draft of the manuscript and prepared the tables. GS and AN prepared the figures. AN revised the manuscript and supervised the study. RM performed the GMDS. AA performed the statistical analysis. All authors contributed to manuscript revision, read, and approved the submitted version.

## Conflict of interest

The authors declare that the research was conducted in the absence of any commercial or financial relationships that could be construed as a potential conflict of interest.

## Publisher's note

All claims expressed in this article are solely those of the authors and do not necessarily represent those of their affiliated organizations, or those of the publisher, the editors and the reviewers. Any product that may be evaluated in this article, or claim that may be made by its manufacturer, is not guaranteed or endorsed by the publisher.
